# Impact of COVID-19 on Food Security in Ethiopia

**DOI:** 10.3390/epidemiologia3020013

**Published:** 2022-03-22

**Authors:** Wenqin Zhang, Léo Persoz, Sandrine Hakiza, Loza Biru, Lemlem Girmatsion

**Affiliations:** 1 Global Studies Institute, University of Geneva, 1205 Geneva, Switzerland; wenqin.zhang@etu.unige.ch (W.Z.); leo-dominique.persoz@etu.unige.ch (L.P.); loza.biru@etu.unige.ch (L.B.); 2 Institute of Global Health, Faculty of Medicine, University of Geneva, 1202 Geneva, Switzerland; lemlem.girmatsion@unige.ch

**Keywords:** healthcare system, food systems, nutrition, food security, Ethiopia, COVID-19, conflict, climate change, food value chain

## Abstract

Since the outbreak of COVID-19, its effects on different aspects of life have been subject to much research, including food security, a domain that has been of special concern in many low-income countries. Ethiopia has been facing many challenges related to food security for decades via drought, famine, and conflict. Within this context, this case study assessed the impact of the COVID-19 pandemic on food security in Ethiopia. Results show that the ongoing pandemic has negatively impacted different regions and at-risk groups in a heterogeneous manner. This has been mainly through disruptions in the Ethiopian food value chain and the relative failure of social security programmes to address the losses generated by COVID-19. The population in the capital city, Addis Ababa, was able to maintain the same level of food security despite income losses caused by the COVID-19 pandemic. However, at-risk groups such as refugees, internally displaced persons (IDPs), and conflict affected regions were seen to suffer significantly from food insecurity exacerbated by COVID-19. Furthermore, this paper particularly emphasizes the importance of considering contextual factors other than COVID-19, such as conflicts or climate change, when discussing the state of food security in Ethiopia.

## 1. Introduction

A novel Coronavirus infection (COVID-19), first identified in late December 2019, was declared a pandemic by the World Health Organisation (WHO) on 11 March 2020. The virus quickly spread, with approximately 456.9 million reported cases and 6.04 million reported deaths worldwide as of mid-March 2022. The impact of this disease is manifold at both national and individual levels, but one of the most important consequences induced by this pandemic is its disruptive effect on food security, especially for the most deprived individuals [1]. Moreover, because food security is the most important aspect of development [2], the impact of COVID-19 on the national food system can be expected to be larger in low-income African countries, such as Ethiopia, which is the subject of this study. Therefore, it seems relevant to assess the specific impact of this global pandemic on the food system of developing countries.

The Constitution of the WHO states that “Health is a state of complete physical, mental and social well-being and not merely the absence of disease or infirmity” [3]. Food security is closely linked with the well-being of people. Food security is achieved when all people always have physical and economic access to sufficient, safe, and nutritious food that meets their dietary needs and food preferences for an active and healthy life [4]. The COVID-19 pandemic has had a significant impact on global food security. It has triggered a recession comparable to that seen after World War II, causing losses in income and disruptions in food supply chains [5]. Naturally, these occurrences affect poor people’s food security more than that of richer people because there is already an imbalance of income and access. People with less tangible income must adjust food choices accordingly, and this effect is stronger with the fewer-income families. Research done by Laborde et al. predicts that for every percentage point of global economic slowdown caused by the pandemic, the number of people living in poverty would increase by 2% to 3% worldwide [5]. The number of people in extreme poverty would then increase by over 140 million, in the absence of effective interventions. This increase in poverty not only reduces access to food but also causes a decline in nutrition and quality of diet [6]. 

COVID-19 has also threatened food security through disruptions in agricultural input markets, farm production, and distribution of food caused by social distancing measures, though in a milder form than through reduced household income [7]. There have been breakdowns in the processing of agricultural products, particularly meat production. This is because low temperatures and proximity of workers can result in very high rates of disease transmission [7]. There has also been a disruption in harvesting, planting, transport, and market exchange because of a lack of workers and seeds, reduced transport facilities, and lockdowns [5]. However, these agricultural disruptions do not impact food security as strongly as reduced income does because global markets are well supplied [8]. 

The pandemic has additionally impacted food security in terms of disruptions to public programmes that provide food, nutrition, and health services. Other community initiatives including nutrition programmes for pregnant women and lactating mothers have also been affected. Through and through, access to food has significant public and global health implications. Lack of food can trigger deficiencies in critical nutrients and calories necessary to fight disease. Additionally, it can result in an increase in consumption of cheaper, nutrient-deficient food, which can bring about health problems such as obesity, diabetes, and hypertension [9].

Building on such foundations, this paper will try to provide an overview of the effects that the COVID-19 pandemic has had on food security and nutrition in Ethiopia. More precisely, it will consist of a case study attempting to investigate the impact of this sanitary situation and government measures on different groups of individual groups present in the country. This part was divided into two main subsections. First, we provided a case presentation of Ethiopia, which, on the one hand, provided general information and the pre-COVID-19 situation related to food security and nutrition in Ethiopia, and on the other hand a description of the health care system’s functioning and the non-pharmaceutical interventions that have been implemented following the COVID-19 aftermath. The case presentation would be helpful to understand why aspects such as the food value chain and different groups in Ethiopia were impacted in a certain way. Second, we turned to a case analysis on some of the consequences of this pandemic for different food-related social security programmes as well as the national food value chains. We then examined the differentiated impact of COVID-19 on affected groups and areas. Finally, the discussion section tried to explain the results of the case analysis, as well as suggest areas of study that could be further explored.

## 2. Materials and Methods

We conducted a real-time analysis of the impact of COVID-19 on food security, as the change is still ongoing along with the pandemic. We collected and used country-specific data from recognized and reliable sources such as government documents from Ethiopia Ministry of Health, United Nations (UN), official World Food Programme (WFP), and Food and Agriculture Organisation of the United Nations (FAO) websites, and published peer reviewed papers.

The time frame we focused the study on is from the first confirmed case in Ethiopia in March 2020 to the last data points in early mid-March 2022. As the humanitarian crisis history of Ethiopia is crucial in the understanding of its current nutrition and food security situation, we included the period prior to COVID-19 in our time frame. For the same reason, we also considered factors such as natural disasters and internal conflicts and included this information in our data gathering. Because the impact of the current pandemic and government actions are ongoing, much of the data and results are still evolving.

## 3. Case Presentation

### 3.1. Agro-Pastoral System, Economic, Political and Geographical Situation

Ethiopia is a land-locked country localized in the Horn of Africa, bordered by Sudan, South Sudan, Eritrea, Somalia, Uganda and Djibouti. Its topography is quite diverse, with high plateaus, mountains and drylands [10]. The uneven distribution of production patterns and agricultural potential in Ethiopia reflects its great geographical diversity. Dega and Weyna Dega zones are the most concentrated areas of agricultural production. Most of the producers in these regions are smallholders, and the production is dominated by five major cereal crops—teff, maize, wheat, sorghum, and barley [11]. The agro and pastoral systems are also unevenly distributed in the country. Most of the Afar, Dire Dawa, and Somali regions rely only on the pastoral system, while central and western Ethiopia mainly rely on cereal production or a mix of cereal and livestock production [12]. Over 80% of the Ethiopian population live in rural areas and are heavily dependent on rain-fed agriculture. Cereals contribute 86% of the total crop production and cover 80% of the cropped land [13]. This makes them extremely vulnerable to changes in weather conditions and has resulted in Ethiopia being dependent on food importation.

Food security and poverty in rural regions are linked with the distance from market towns. Poverty rates increased by 7% with every additional 10 km distance from a market town of at least 50,000 people [4]. This means that rural households living far from towns are less likely to have access to tools for agricultural growth and are therefore more likely to be food insecure. Pastoral regions such as Somali, Afar, and parts of Oromia are more vulnerable than other regions of the country due to poor infrastructure, lack of market accessibility, and livestock-based livelihood [4]. The Tigray, Afar, and Somali regions have been reported as the ones most often experiencing drought. In years when these regions do not receive sufficient rainfall, even if national food production totals are unaffected, these regions are unable to access food through the market [14]. Therefore, whilst there are periods when there are no dips in national rainfall totals, local drought incidences in the East and Northeast regions of the country still have serious impacts on livelihoods, leaving the local populations unable to meet their food needs by buying from other regions [14].

As climate change becomes more and more of a reality, changes in rainfall amounts and the increasing frequency and intensity of droughts will lead Ethiopia, a country that has contributed little to these changes, to become a more food insecure country [14]. Rising temperature and variations in rainfall create problems for pastoralists who live in already dry areas, and these populations are now switching from cattle to goats and camels as they are more able to endure long periods of droughts [15]. In the central part of the country, more rain will mean increased soil erosion and decreased crop yields for smallholder farmers who generate 95% of the total agricultural production in the country, while low lying areas will suffer from floods [13]. Increasing temperatures and rains attributed to climate change in desert regions also impact food security by providing new environments for pest breeding, development, and migration [15]. East Africa’s worst desert locust upsurge in at least 25 years threatened crops and pastures across the region, particularly in Ethiopia, Kenya, and Somalia [16]. The worst affected region was Oromia, followed by the Somali and Tigray region, which are already chronically food insecure [17].

Displacement is another key factor of food insecurity. In recent years, conflict, climate change-induced natural disasters, and planned resettlement programmes have resulted in the displacement of over 2 million people [18]. Communities that are displaced most often lose their productive assets such as homes, domestic animals, and crops, which in turn disrupts their access to food as well as clean water [18]. Additionally, when internally displaced peoples are introduced to host communities, these communities also face increased pressures in terms of food security [18]. 

Parallel to the effects of displacement on food security, armed conflict also plays a major role in the accessibility to food in Ethiopia. From wars of independence to the Ethiopian civil war of 1974 to the ongoing Oromo and Tigray conflict (which are motivated by complex intricacies of ethnic, land and political divergences [19]), armed struggle is, regrettably, weaved into the fabric of Ethiopia. Conflicts disrupt markets, trade, and crop production; prevent herders from accessing their pasturelands; and contribute to increased food prices [20]. Moreover, crops are employed as tactics of war because intentional use of starvation is conducted through deliberate destruction of agriculture and food production capacity [21].

In terms of determinants of food security in Ethiopia, a study by Abafita and Kim found that households with higher food security had a higher mean per capita consumption expenditure, more literate heads of households, adequate rainfall, and more livestock. These households were also more likely to be smaller in number and to participate in off-farm activities [22]. Surprisingly, the study also found that households with access to credit and remittance receipt were more likely to be food insecure, possibly because funds were not being used productively. Additional studies found that adoption of yield-enhancing technologies such as irrigation schemes, improved crop and livestock varieties, use of fertilizers, and solid and water conservation had positive effects on food security [23].

Regarding economic matters, the general performance of Ethiopia is rather positive. For instance, the national real Gross Domestic Product (GDP) was around 249 billion US dollars in 2019, for annual growth of 6.68% [24,25]. However, this general positive performance of the Ethiopian economy seems to have benefited the Ethiopian population in a contrasting fashion. Although the World Bank reported that poverty decreased in both rural and urban areas between 2011 and 2016, this decrease has been more impactful in the latter group—during this period, urban areas saw a decrease in poverty of over 10%, while rural areas experienced a reduction of less than 5% [26]. Moreover, by relying on a multivariable analysis involving monetary as well as non-monetary indicators to account for household deprivation, Bersisa and Heshmati (2021) reported a poverty incidence of approximately 80% in small towns and rural areas. The definition of poverty used in this study follows the multidimensional poverty measures suggested by the Alkire Foster method, which accounts for deprivation and capabilities and not only expenditure and income [27]. This uneven economic situation has created disparities regarding access to different health services, such as protection against certain infectious diseases or healthcare for children under five, and drives food insecurity and malnutrition [28,29].

In 2014, Ethiopia was ranked first in having the highest number of people in a state of undernourishment by the Africa Food Security and Hunger Multiple Indicator Scorecard. The number was 32.1 million people [4]. Food insecurity in Ethiopia, both before and during the COVID-19 pandemic, is highly linked but not limited to severe food shortage and famine caused by recurring drought [4]. Displacement, desert locusts, climate change, and conflict are additional aggravating factors for food insecurity in Ethiopia. About 10% of Ethiopia’s population are chronically food insecure, and this number increases to more than 15% during frequent drought years [4].

### 3.2. Healthcare System

Since the 1970s, Ethiopia has been implementing a primary healthcare approach that has focused on disease prevention and control, given priority to rural areas, and advocated for community involvement [30]. The current healthcare system was implemented in 1993, with increasing access to primary healthcare services, and consists of primary hospitals, health centres, and health posts. Primary hospitals provide promotive, preventive, curative, and rehabilitative outpatient care; basic emergency surgical procedures; and comprehensive emergency obstetric care. Health centres provide promotive, preventive, curative, and rehabilitative outpatient care, and inpatient care and health posts provide essential promotive and preventive services and limited curative services.

From 1995 to 2015, the Ethiopian government developed and implemented the first 20-year health sector development programme in four phases [30]. These phases included advancements such as improved access to and quality of health services, improved health care financing, creation of the health extension programme, enhanced pharmaceutical services, and improved health infrastructure. With the launching of the Health Extension Program (HEP) in 2003, Ethiopia was able to achieve significant improvements in maternal and child health, prevention and control of communicable diseases, increases in hygiene and sanitation, healthcare education and communication, and community engagement. The government introduced its second 20-year strategy of strengthening primary health care in 2015. This plan aligns with Sustainable Development Goal (SDG) 3 and aims to build the health system capacity, improving universal health coverage. The current phase aims to expand coverage of services for noncommunicable diseases and mental health [31].

**Figure 1:**
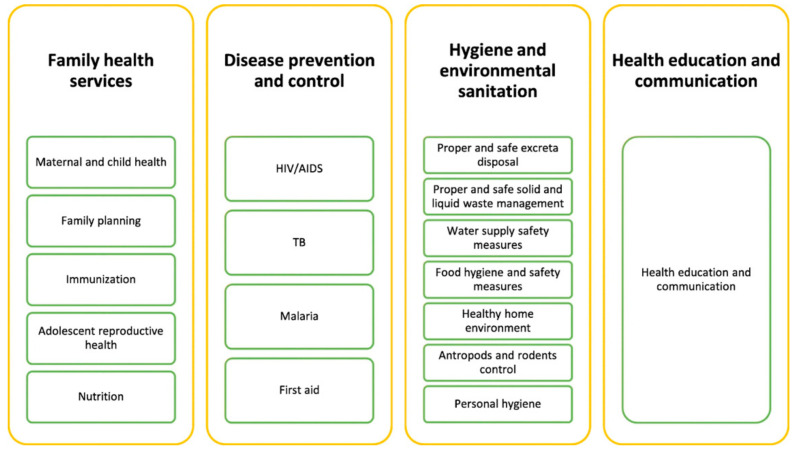
Packages of the rural health extension program of Ethiopia [32].

The HEP became operational in 2004–2005 with the graduation of 7136 Health Extension Workers (HEW), trained to work mainly in disease prevention and health promotion in rural villages [32]. Nutrition is targeted under the family health services section of the health extension program (Figure 1) [32]. The program promotes community nutrition by collecting appropriate information for preparing nutrition education. It provides basic nutrition information and education such as preparation and feeding of nutritional foods to community members. Nutritional needs of pregnant and lactating women are also a focus of the family health services. Health extension workers are taught how to check for signs and symptoms of malnutrition and undernutrition [33]. Despite great achievements in maintaining essential health services, the pandemic led to an increase in undernutrition, particularly among children under five. This was due to declines in household incomes; changes in the availability and affordability of nutritious food; and interruptions to health, nutrition, and social protection [34].

### 3.3. COVID-19 Related Epidemiological Situation and Non-Pharmaceutical Intervention

While the first case of COVID-19 was reported in Wuhan China in late December 2019, Ethiopia did not declare its first COVID-19 case until 13 March 2020 [35]. This first identified case was a Japanese traveller returning to Ethiopia from Burkina Faso on 4 March 2020. Currently, Ethiopia is the most COVID-19 affected country in East Africa and the fourth most affected on the continent [35]. As of 11 March 2022, the total number of reported COVID-19 cases in Ethiopia stood at 469,153, 40,234 of which were active. Since the start of the pandemic, 7484 COVID-19-related deaths have been recorded [36]. Testing is low in the country, with a cumulative of around 4.5 million tests, including both PCR and antigenic tests, undertaken since the start of the pandemic [35]. This suggests that most COVID-19 cases are unreported.

**Figure 2:**
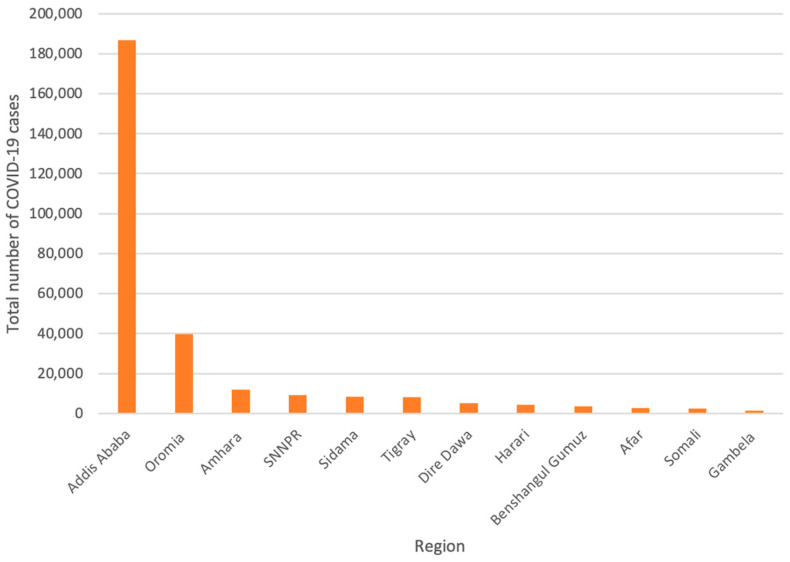
Confirmed COVID-19 cases across Ethiopia’s regions [36] Adapted from Ethiopian Public Health Institute. Covid 19—Ethiopian Public Health Institute. 2021. Available online: https://ephi.gov.et/ (accessed on 17 March 2022).

Ethiopia’s urban areas have the highest burden of COVID-19 in the country. Out of these urban areas, the capital city, Addis Ababa, remains the hardest hit with a confirmed case number of 186,937 and a death toll of 122 (Figure 2) [36].

In terms of sex distribution, most of the cases were seen to occur in men (60.69%), within the ages of 20 to 30 [36]. Early in the pandemic, the reported COVID-19 cases were mainly imported by travellers. However, as the pandemic has progressed, community spread has taken over, becoming responsible for 50.68% of the reported COVID-19 cases in Ethiopia [35]. 

The COVID-19 vaccine campaign, in Ethiopia, started on the 13 March 2021. This was following the delivery of the first 2.2 million doses of the AstraZeneca vaccine to the country on the 4 March 2021, through the COVAX initiative [37]. The AstraZeneca, Sinopharm and Johnson and Johnson vaccines are the main COVID-19 vaccines currently in use in the country. While the original aim was to vaccinate 20% of the population by the end of 2021, currently only 2.6% of the population is partially vaccinated due to lack of availability of the vaccine [38].

### 3.4. Non-Pharmaceutical Interventions

**Figure 3:**
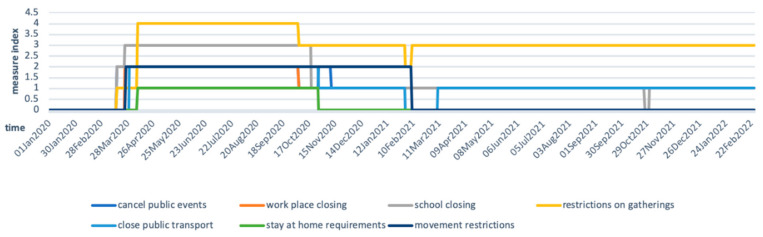
Ethiopian government measures on containing COVID-19. Adapted from: Hale, Thomas, Sam Webster, Anna Petherick, Toby Phillips, and Beatriz Kira (2020). Oxford COVID-19 Government Response Tracker, Blavatnik School of Government. Data use policy: Creative Commons Attribution CC BY standard [39].

Since Ethiopia’s first COVID-19 case on 13 March 2020, multi-sectoral measures have been taken by authorities, including closing public transport, banning public gatherings and other social activities, and school closures for several months. Oxford COVID-19 Government Response Tracker collects data daily to track government’s response to COVID-19 [39]. The scores are calculated based on the intensity of the measurement and its geographical scale. The higher the score is, the stricter and wider the measurement applies. Figure 3 shows that the Ethiopian government started to impose strict measures, including closing public spaces and restricting gatherings, from April 2020. Measures were gradually relaxed in October 2020 and February 2021. Stay-at-home requirements and movement restrictions were lifted in late October 2020 and February 2021, respectively, while strong measurements on gathering restrictions remained over time.

Movement restrictions were implemented for a time after the first case, including mandatory 14-day quarantine for travellers coming from outside of Ethiopia, land border closures, and prohibition of all non-essential inbound and outbound traffic. Tigray, Oromia, Amhara, and several other regional authorities also banned public transportation and put restrictions on other vehicle movements between urban and rural areas [40]. *The Protocol for Screening and Quarantine of Cross-Country Truck Drivers and Other Crews* was issued on 8 May 2020, to enhance the health screening, testing, case management for truck drivers, and decontamination of trucks at the point of entry and final destinations.

Contact tracing was also adopted to interrupt chains of transmission by listing, tracing, and following-up persons who had contact with confirmed COVID-19 cases. According to data from the Ministry of Health of Ethiopia, as of 11 July 2021, a total of 374,083 contacts of confirmed cases had been identified, and 87.95% of the contacts had completed 14 days of follow-up. 11.27% of the contacts had tested positive, and this contributed to 15.22% of the total cases [41]. Health screening at the points of entry and follow-up of international travellers have been conducted to control cases coming from outside of Ethiopia. Antigen Rapid Diagnostic Testing (Ag-RDT) for COVID-19 started at Bole International Airport for returnees on 7 July 2021.

Since Ethiopia had already established a social protection system, namely, the Productive Safety Net Program (PSNP), before the pandemic, after the outbreak of COVID-19 [42], the Ethiopian government was able to quickly scale up social assistance based on the original system. Direct support continued during COVID-19, and for the public works programme, work requirements were waived for payment during the lockdown. In the Multi-Sectoral Preparedness and Response Plan developed by the government, three vulnerable groups of urban households were recognized for support delivery through the Urban Productive Safety Net Program (UPSNP). Besides cash transfers to existing UPSNP direct support beneficiaries, unregistered poor urban households in the UPSNP cities and new urban centres received 3 months of unconditional cash transfers. Using community-based targeting, more households were registered despite the movement and public gathering restrictions [42].

Positive results have been observed among PSNP participants compared to non-PSNP participants [43]. However, there is a limitation on the coverage of PSNP. Only 11 major cities in Ethiopia are covered by the programme, and for the 86.5 million people living in rural areas [44], only 7 to 8 million receive PSNP assistance, which accounts for less than 10% of the rural population [45]. Studies assessing more precisely the PSNP’s performance during the pandemic will be presented further.

## 4. Impact of COVID-19 on Food Security in ETHIOPIA

### 4.1. COVID-19 Impact on Food Value Chain

COVID-19 has disrupted the food value chain through factor market, production, transportation, and buying behaviours, further influencing food price and availability negatively. Prices reflecting supply and demand of different commodities further impact households from urban, rural, and different regions in Ethiopia.

**Figure 4:**
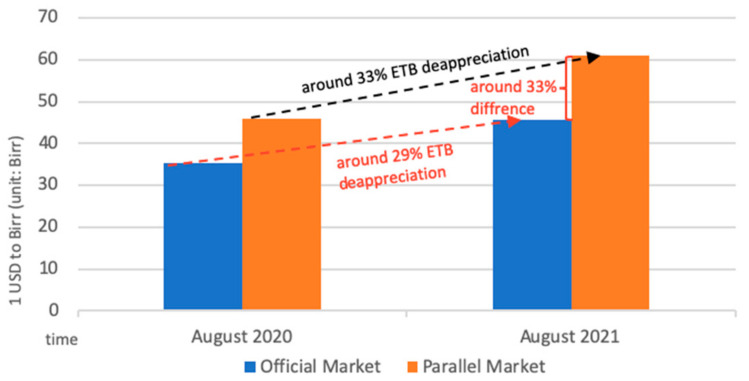
Official and parallel market exchange rate of United States Dollar (USD) to Birr. Adapted from WFP. *Monthly Market Watch Ethiopia|August 2021*; World Food Programme: Addis Ababa, Ethiopia, 2021.

**Figure 5:**
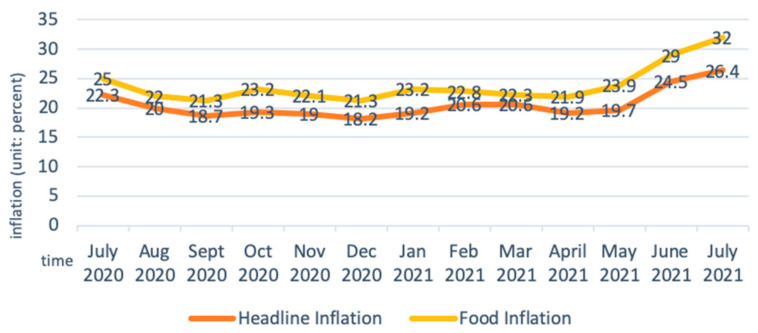
Headline and food inflation in Ethiopia. Adapted from: Anon (n.d.). Ethiopia Food Inflation|2021 Data|2022 Forecast|2013–2020 Historical|Chart. Available online: https://tradingeconomics.com/ethiopia/food-inflation.

The price of agricultural inputs has surged, and accessibility has deteriorated amid the pandemic due to the currency (Ethiopian Birr, ETB) depreciation and a surge in inflation. From August 2020 to August 2021, the exchange rate of Birr to the US dollar fell sharply in both the official and parallel markets. The decline in the parallel market was even greater than the official market, leading to a large differential between the two markets (Figure 4) [46]. The Somali region, which relies on informal imports of pasta, rice, and wheat flour as staple cereals, is particularly affected by the currency depreciation in the parallel market. Food inflation largely contributed to the headline inflation, which in July 2021 reached the highest point in nine years (Figure 5) [47]. Prices of oils, fats, cereals, milk, and dairy products had the highest surge [46], putting high pressure on rural and urban households that are market dependent. Inflation and currency depreciation also raised the domestic and import price for agriculture inputs, such as fungicides, insecticides, herbicides, fertilizers and improved seeds, which are crucial inputs for vegetable production [48].

Studies have shown that the impact of COVID-19 restriction measures on the food value chain varies depending on the characteristics of a commodity, including the resource needed for production and its intensity [5]. Agriculture in Ethiopia is labour-intensive, and although small holder farmers account for most of the Ethiopian agricultural economy, medium-large scale farmers and the gig economy actors in commercial agrarian settings have been emerging in recent years [49]. Flexible contract arrangements are one feature of these agro-economies. The vegetable production in the Central Rift Valley, for example, attracts many daily workers. The laborers usually gather at certain locations in rural towns to be picked up by the producers [48]. 

As local and migratory laborers are disrupted by border closure, movement, and gathering restrictions, productions of different food are below average. Large commercial farms along the Awash River in Afar; Humera areas of Tigray; Metama in Amhara; and parts of Oromia and Southern Nations, Nationalities, and Peoples’ Region (SNNPR) that depend on migratory laborers are notably impacted [50]. Despite the favorable Kiremt rainfall in 2021, land preparation and planting of short-maturing crops are below average in parts of central and eastern Oromia and SNNPR along the Rift Valley [51].

Even though livestock price has increased despite the declined body condition across many pastoral areas due to pasture and water shortage, as staple food prices increase more significantly, the terms of trade (TOT) disfavours pastoral and agro-pastoral households. According to the Somali Disaster Prevention and Preparedness Office, the goat to maize TOT in the Jigjiga market in July 2021 was over 30% lower than the five-year average [51]. The unstable and inconsistent price fluctuations of staple food and animal products have led to a decline in the purchasing power of pastoralists, which further impacts the food security of households depending on pasture and livestock income. 

Farmers’ access to input and output markets are limited by reduced food commodity movement due to transportation impacted by travel bans, border testing requirements, and high transportation cost. The authority issued the *Protocol for Screening and Quarantine of Cross-Country Truck Drivers and Other Crews* on 8 May 2020. Measures such as thermal and clinical health screening at point of entry, decontamination of trucks at point of entry, and destination prolonged transportation time, producing further delays in the supply chain. Travel bans have also reduced the volume and frequency of trucks running between areas. 

Surveys taken by the International Food Policy Research Institute (IFPRI) on the vegetable and dairy value chains in Ethiopia in 2020 show that fear of disease risk and the slowdown of restaurant businesses resulted in falling consumption of vegetable and dairy products. Consumers linked raw milk, vegetables, and their downstream products with a higher risk of contracting the virus and perceived those traders who had contacted many people and travelled to different places in the distribution process having high COVID-19 risk. Some farmers interviewed said that they had to leave some vegetables to rot in the field due to the lack of buyers [48,52].

**Figure 6:**
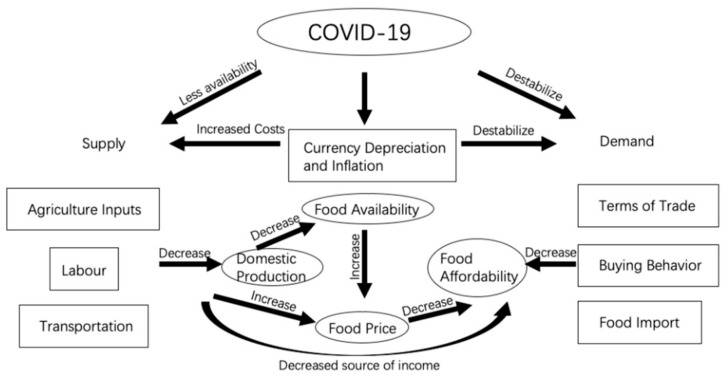
How COVID-19 impacts the food value chain in Ethiopia.

As a country with high sensitivity and vulnerability, the food value chain in Ethiopia has been hit harder by COVID-19 than countries with a robust food system (Figure 6). Sensitivity and vulnerability are intertwined as some of the regions in Ethiopia highly depend on import of food input and staple, even foreign labours; the food value chain is sensitive to fluctuation in the foreign exchange and petrol prices and its ability to find alternatives is limited. Thus, disruption of any link in the food value chain will magnify the impact on food prices and availability. Considering that the food value chain is the source of income for many households in Ethiopia, food affordability will become even more problematic.

### 4.2. Ongoing and Disrupted Food-Related Social Security Programmes

This chapter aims to examine the added effects of the COVID-19 pandemic on food security in Ethiopia. This will be accomplished by reporting the degree to which the domestic public programmes related to food security have managed to mitigate the adverse effects of COVID-19 on Ethiopian households. As introduced in the case presentation, the PSNP is widely seen as the flagship of the safety net in Ethiopia. Therefore, assessing the performance of this programme is of primary importance regarding the purpose of this section and this article.

To this end, an evaluation of the PSNP from March to June 2020 found that households targeted by the PSNP were less prone to being food-insecure during the beginning of the COVID-19 pandemic than the ones who did not benefit from the PSNP [43]. By relying on data obtained through a pre-pandemic, in-person household survey and a post-pandemic phone survey, this study found that the COVID-19 and its consequences have left half of the households included in the study more food insecure than the pre-pandemic period. Nevertheless, the beneficiaries of the PSNP have reported relatively less deterioration than the non-beneficiaries. Moreover, the poorer and more remote households that benefit from the PSNP have been provided with slightly more help than the others. More practically, the PSNP appears to reduce the likelihood of households adopting coping strategies as a result of the COVID-19 aftermath [43].

However, other studies present less optimistic results. Study conducted by Deshpande et al. found that although the government managed to provide relatively more assistance to the poorest share of its population compared to the richest (in this study, the population has been divided into two subgroups, the richest one (60% wealthiest Ethiopian households) and the poorest one (the remaining 40% of Ethiopian households, considered as the poorest ones) [53]), only 13% of the population had received some form of assistance as of October 2020 [53]. Moreover, there was a huge gap between households that had experienced a reduction of income (74.8%) and households that had received some form of assistance (13%) between mid-March and mid-October. Besides, the governmental help did not exceed $45 per month between March and October, and some form of assistance came only once during this period for 57.7% of the poorest households that had benefited from assistance [53]. Thus, even though it can be concluded that the authorities have managed to target low-income families as stipulated earlier, this assistance was not only quantitatively limited but also failed to be distributed to most parts of the households that reported reduced income following the COVID-19 aftermath. This shows that even though, as mentioned above, the PSNP can be considered to some extent as effective at mitigating the consequences of COVID-19 for the household it helps, it does not follow in any case that the Ethiopian authorities have managed to aid most of the population that have been negatively affected by the pandemic.

Furthermore, it must also be noted that the government has not managed to maintain some of its social protection programmes after COVID-19 pandemic struck the country. For instance, the school feeding programme running in Addis Ababa has been interrupted following the closure of schools [54]. As school is the main meal of the day for most children from low-income families, this has had important consequences on children covered by this programme as well as their family. Indeed, by conducting 53 in-depth qualitative interviews, Delbiso et al. found that this school feeding programme’s interruption has caused not only food shortages and meal skipping—coping strategies that are already commonly used during food security crisis [55]—but also child abuse as well as child labor [54]. To compensate for such examples of the negative consequences that can follow from school feeding programmes’ closure, it can be noted that during the months of June, July, and August 2020, the WFP has provided take-home rations to approximately 272,000 children’s families throughout Ethiopia [56]. 

What should be retained from this section is that, overall, Ethiopia has struggled to ensure the adequate functioning of its food social security programmes in a time where food insecurity has increased throughout the country. Indeed, while the PSNP has managed to provide help to the individuals it already covered before the COVID-19 pandemic hit the country, it has mostly failed to compensate for the increased number of individuals at risk of being food insecure following the COVID-19 aftermath. Moreover, the government has not managed to maintain the running of other food related social security programmes, such as the school feeding programmes in Addis Ababa. Suggestions explaining this rather complicated situation will be provided in the discussion section.

### 4.3. Differentiated Impacts on Affected Groups/Areas

#### 4.3.1. Urban Areas

The COVID-19 pandemic has disproportionately affected the urban parts of Ethiopia, due to their high population density. Those employed in informal sectors; retail; hospitality; and micro, small, and medium-sized enterprises were the hardest hit by the economic shutdown caused by the COVID-19 pandemic [57]. However, the effects of the economic shutdown on food insecurity varied in the different urban areas of Ethiopia. 

Addis Ababa, the capital city of Ethiopia, and the city with the heaviest burden of COVID-19 cases, is an interesting example. Due to the large variations in wealth status found in this city, both cases of overnutrition and undernutrition were observed even before the pandemic. Undernourishment was mainly seen in children, affecting 15% of children under five within the city [58].

However, a study assessing a representative sample of households in Addis Ababa, through the conduct of telephone surveys, found that food insecurity had not changed significantly from what was reported before the pandemic, in September 2019. Despite reported income losses in 64% of interviewed household, as a result of COVID-19 restrictive measures, these households still managed to maintain a similar level of food consumption. While a significant impact on food consumption was not observed, the composition of the types of food consumed did change. The surveyed households in Addis Ababa were seen to move more towards staple foods while avoiding vegetables and raw meat, reducing their nutritional diversity. However, this move away from raw meat and vegetables may be a result of beliefs that they carry COVID-19 risks rather than the unavailability of these food items [59].

These unexpected results suggest that food security and consumption has remained resilient despite the financial challenges posed by the COVID-19 pandemic in Addis Ababa. Although there is a lack of literature explaining this resilience, potential reasons could be that expenditures on non-food items may have now been redirected to food items with the closure of bars or cinemas early in the pandemic [59]. Moreover, a previous study surveying the same households found that many households used cash or bank savings in order to cope with income loss, which may have allowed for the maintenance of consistent food consumption [60].

**Figure 7:**
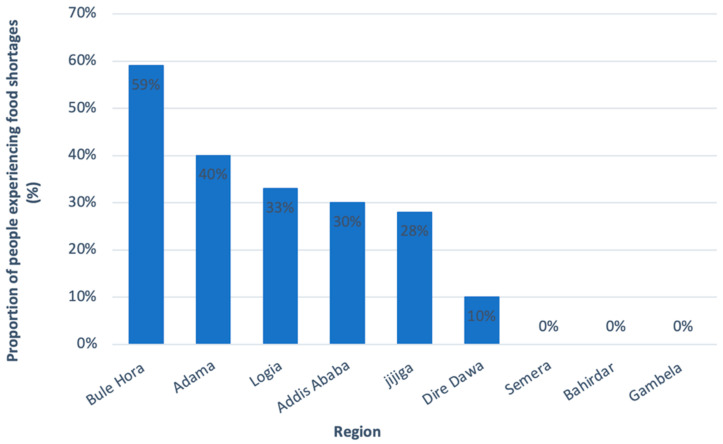
Incidences of food shortages, amongst the most vulnerable groups (UPSNP recipients, small scale business owners, IDPs, and refugees), in the nine main urban regions of Ethiopia. Adapted from Harris, D. et al. The Effect of COVID-19 and Government Response Measures on Poor and Vulnerable Groups in Urban Areas in Ethiopia; Oxford Policy Management: London, UK, 2021 [61].

Despite the resilience of Addis Ababa’s food security, households with a lower wealth status, and households in urban areas outside of Addis Ababa, faced a greater risk of food insecurity resulting from COVID-19. This risk is reflected in a study assessing vulnerable households in nine different urban areas of Ethiopia since the start of the pandemic. Within these vulnerable groups, 27% of beneficiaries of the UPSNP, despite receiving assistance, reported having experienced food shortages. Increased food prices, devaluation of the Ethiopian Birr, and an already low income following the pandemic were cited as factors leading to food insecurity. Workers in the informal sector, daily wage earners, daytime laborers, street vendors, and women were amongst the most vulnerable to income loss and hence food insecurity [61]. In addition, food shortages were reportedly more severe in smaller cities such as Bule Hora and Adama (Figure 7). Nonetheless, the study has found that as the pandemic progressed, food security began to recover back to pre-COVID-19 levels. This suggests that even among the most vulnerable groups in urban areas, COVID-19-related food insecurities were just a temporary shock as opposed to a more permanent issue [61].

#### 4.3.2. Rural Areas

While Ethiopia’s urban areas had the heaviest share of COVID-19 cases, the economic shutdown caused by COVID-19 restrictions still had a significant impact on food security in rural areas. Eighty percent of the Ethiopian population resides in rural areas, with agriculture as the main source of income and economic activity. In a study by the world bank, loss of income was reported in 25% of farming households at the beginning of the pandemic. Moreover, some households reported that they were unable to maintain their normal level of farming activities since the start of the pandemic, citing the inability to hire labor, stay-at-home advice, and mobility restrictions as the main reasons. As most Ethiopian farmers are subsistence farmers, this reduction in farming activity can also result in food insecurity [62]. 

While most urban households relied on savings to cope with income losses related to COVID-19, the same was not true for rural households. In the rural households studied by the world bank, 60% could not cope with the loss of income at all [62]. Being less likely to adapt to income shocks, such as the one caused by the pandemic, rural households faced a greater risk of food insecurity. Based on telephone surveys conducted in late 2020, increased food prices and income loss, as a result of COVID-19 restrictions, were cited as main reasons for food insecurity. In these same rural household surveys, 46% of respondents reported having difficulties in obtaining food from markets. Another 14% responded saying that they had seen a significant decrease in food availability in markets, possibly caused by farming disruptions. Moreover, 48% of rural households were reported to be experiencing moderate to severe food insecurity in May and June of 2020 [62].

While the impacts of COVID-19 were apparent at the beginning of the pandemic, these effects were seen to taper off as the pandemic progressed and households seemed to adapt. In addition, while movement restrictions did affect economic activity in rural households by reducing daily laborers, for the most part, the households themselves were able to maintain farming activity. It is also important to note that in 2020, the locust invasion affected food security and farming outcomes beyond COVID-19 [62].

#### 4.3.3. IDPs and Refugees

As mentioned above, households already vulnerable to food insecurity are more prone to face further challenges as a result of COVID-19. Refugees and internally displaced people (IDP) are amongst the most vulnerable groups to food insecurity, usually forced to rely on aid and food assistance [63]. According to the United Nations High Commissioner for Refugees (UNHCR), refugees in Ethiopia were at risk of food insecurity and malnutrition even before the COVID-19 pandemic. In a 2018 report, 91.3% of the camps surveyed presented global acute malnutrition. As a result, a significant number of children from these refugee camps have had to be treated for malnutrition [63].

Ethiopia is currently the third largest refugee-hosting country in Africa. Most of the registered refugees in Ethiopia come from its neighbouring countries: South Sudan, Somalia, Eritrea, and Sudan [64]. In addition to this, Ethiopia also holds a large population of IDPs, resulting from ethnic and border conflicts. An International Organisation for Migration (IOM) report estimated that over 4 million people were displaced nationwide during June–July 2021 [65].

According to the ninth Ethiopian national displacement report, IDPs and returnee IDPs were found to be amongst the most affected groups, in Ethiopia, by the COVID-19 pandemic. The supply of products, including food, decreased while the price of food was rising. Shortage of food, as assessed by this report, was present in 81% of the villages housing returning IDPs. In addition, 85% of the assessed villages reported that food prices had increased, affecting their ability to purchase food items [65]. Similarly, increase in food prices and general shortage of food supply were also noted among current IDPs. Eighty-one percent of IDPs in the assessed sites mentioned that they were experiencing food shortage, while food prices were seen to increase in 83% of the sites. 

Moreover, a mixed method panel study on IDPs and refugees sheds light on the impact of COVID-19 in these groups. The devaluation of the Ethiopian Birr, reduction of income, and an increase in prices of food following the start of the COVID-19 pandemic were identified as exacerbators of food insecurity. Eating less frequently and avoiding the purchase of expensive foods were reported as coping methods by IDPs and refugees. While staples such as white flour were the highest in their diets, eggs, meat, and dairy products were reduced or completely avoided by these households. Moreover, a study by Harris et al. (2021) reported food shortages were much higher in the IDP and refugee households than in the other low-income households assessed [61].

IDPs and refugees, already at risk to food insecurity, are currently facing greater challenges due to the COVID-19 pandemic [57]. However, it must also be noted that the recent conflict in the northern regions of Ethiopia has further heightened food insecurity, having displaced a large population from their homes.

#### 4.3.4. Conflict Located Areas

**Figure 8:**
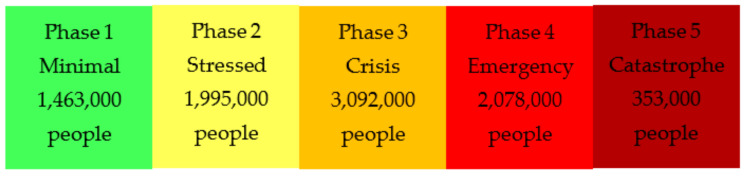
Integrated food security phase classification (IPC) of the 9 million people analyzed in northern Ethiopia. Adapted from IPC. Ethiopia: Acute Food Insecurity Situation May–June 2021 and Projection for July–September 2021. 2021. Available online: https://www.ipcinfo.org/ipc-country-analysis/details-map/en/c/1154897/ (accessed on 17 March 2022) [67].

The northern Tigray, Amhara, and Afar regions, where the current conflict is mainly concentrated, face the highest level of food insecurity within Ethiopia [66]. Food security already impacted, both in the rural and urban areas of these regions, by the COVID-19 pandemic has been exacerbated further by the ongoing conflict [67]. Analysis done by the IPC (Integrated Food Security Phase Classification) on these three regions’ acute food insecurity situation between May–June 2021, as well as projections for July–September 2021, estimated that 5.5 million people face food insecurity [66]. Of the estimated 5.5 million people in these regions, over 350,000 people were already in Catastrophe phase (IPC Phase 5), indicating famine (Figure 8) [66]. This is the highest number of people in this phase since the famine in Somalia in 2011 [66].

To summarize, Ethiopia’s food security has been threatened and exacerbated by the COVID-19 pandemic in different ways and has had a varied impact across the country. Ethiopia’s sensitive food value chain was hit hard by COVID-19, with its impacts reflected in increased food prices and shortage of food availability. While social protection programmes such as the PNSP exist to counter these impacts, the Ethiopian government was unable to keep up with the increased level food insecurity that followed COVID-19. As a result, high risk populations such as beneficiaries of PNSP, refugees, and IDPs reported increased food insecurity. Additionally, the recent conflict Ethiopia’s northern region has added an additional strain to Ethiopia’s food security that had already been impacted by COVID-19.

## 5. Discussion

Food insecurity in Ethiopia is already exacerbated by many different factors. Recurring droughts, though often not national events, cause severe damage to regional access to agriculture in the east and Northeast. Desert locusts, recently propagated by extraordinary rains due to climate change, can cause damage to enormous portions of crop fields. Conflict and ensuing displacement leave hundreds of thousands of people vulnerable to food insecurity by way of loss of homes and domestic animals, lack of access to clean water, and interrupted access to markets and trading. It is important for these factors to be considered when examining the impact of COVID-19 on food security in Ethiopia.

Food value chain has two implications for the food security of a country, namely, the food supply, and a source of income that determines households’ purchasing power. A resilient food system involves not only food but also the robustness of a country’s currency and market, its dependence on imports and exports, and the social security system. In the case of Ethiopia, due to its dependency on imports and exports, the price surge in imported staple foods, fuel, and agricultural input caused by the depreciation of ETB has put greater pressure on both producers and consumers. As the agricultural field is labor-intensive, food production activities are disrupted by the lack of labor when travel restrictions are implemented, resulting in a shortage of food supplies, rising food prices, and severe economic hardship for poor families who count on labor as a source of income. 

Compared with other regions, the pastoral regions of Ethiopia (Somali, Afar, and parts of Oromia) have been hit harder. The poor infrastructure condition, droughts, rising temperatures, and desert locust situation, which were particularly pronounced in these regions before COVID-19, have already put these areas in a vulnerable position, making regions highly dependent on food imports. The inconsistency in price growth of staple and animal products, and greater currency depreciation in the parallel market after COVID-19, have produced greater food security challenges to pastoralist households and caused further imbalances among poor pastoral regions and other parts of Ethiopia. 

Regarding ongoing and disrupted domestic social security programmes, we have reported above that the PSNP and other programmes have struggled to support many individuals who became more prone to food insecurity amid the COVID-19 pandemic. This problem might be partly explained by the fact that the pandemic is not the only concern for Ethiopian authorities. For instance, as the ongoing conflicts in Oromia and Tigray have intensified since mid-2020, it seems that the Ethiopian authorities have been more actively working on dealing with this conflict rather than with the COVID-19 pandemic and its impact on food security and nutrition. This idea has been suggested by different authors, who have claimed that most of the government and public’s attention has been centred on the war in Tigray [52]. Without further investigation, it is not possible to make a firm causal conclusion concerning the current situation related to the pandemic in Ethiopia, but if the different conflicts currently raging in certain regions of Ethiopia continue to take place, it seems complicated for the Ethiopian Government to efficiently deal with the adverse impact of COVID-19 on food security. 

Finally, while the COVID-19 pandemic has negatively impacted Ethiopia’s food security, further research needs to be conducted to identify these impacts in specific households. Currently, most studies are based on surveys and self-reported answers concerning food security. These answers could potentially be affected by a social desirability bias. Moreover, due to the social distancing measures resulting from the pandemic, many of the studies collected their data through phone surveys. This poses a problem since phones are generally only accessible to wealthier households, particularly in rural households. As a result, some of the households most vulnerable to food insecurity may be getting overlooked. Furthermore, due to the conflict in the northern region of Ethiopia, data from the most affected regions, particularly Tigray, are scarce. Therefore, it is currently difficult to get a holistic image of COVID-19′s impact on food security and nutrition in the whole country as some regions remain inaccessible and excluded.

## Data Availability

All data used in the presented research work are publicly available.
